# Primary health care reforms in Estonia: using financial incentives to encourage multidisciplinary care

**DOI:** 10.1093/eurpub/ckac129.625

**Published:** 2022-10-25

**Authors:** T Habicht, K Kasekamp, E Webb

**Affiliations:** 1 WHO Barcelona Office for Health Systems Financing, WHO, Barcelona, Spain; 2 Institute of Family Medicine & Public Health, University of Tartu, Tartu, Estonia; 3 Department of Health Care Management, Technical University Berlin, Berlin, Germany; 4 European Observatory on Health Systems and Policies, Technical University Berlin, Berlin, Germany

## Abstract

Estonia has a historical legacy of large hospital networks and municipality-owned specialist clinics, with a low emphasis on primary health care (PHC). Since the 1990s, a transition towards PHC has occurred, delivering PHC in family physician practices rather than in specialist clinics. The transition has been underpinned by a series of comprehensive healthcare system reforms starting in the late 1990s. The most recent reforms, although lacking a legal basis, have been accompanied by financial incentives including EU structural funds to encourage change. These financial incentives were designed to improve quality of care, encourage working in remote areas, and more. A key focus of PHC reforms has been an emphasis on multidisciplinary care, and the reforms have aimed at increasing the involvement of home nurses, midwives, and physiotherapists in PHC. The reforms have also prioritized PHC centres, with multiple practicing physicians, over single physician practices. Although EU structural funds have supported building the infrastructure for expanded scope of services at PHC level, the uncertainty of long-term funding of expanded services remained a key challenge limiting the success of the reform. Further, the supply of family physicians will be problematic in the future, as the number of permanently vacant positions has quadrupled in the last five years and almost half are 60 years of age or older. As the PHC reform process in Estonia continues until today, it can serve as a case study for other countries interested in strengthening their PHC systems.

